# A Solidified Controllable Resin System Suitable for Fracture Cavity Formation Plugging and Its Performance Characterization

**DOI:** 10.3390/gels10090599

**Published:** 2024-09-20

**Authors:** Shuanggui Li, Biao Qi, Qitao Zhang, Jingbin Yang

**Affiliations:** 1 Key Laboratory of Enhanced Oil Recovery in Carbonate Fractured-Vuggy Reservoirs, SINOPEC, Urumqi 830011, China; 2 SINOPEC Northwest Company of China Petroleum and Chemical Corporation, Urumqi 830011, China; 3 School of Petroleum Engineering, China University of Petroleum (East China), Qingdao 266580, China

**Keywords:** fracture-cavity formation, resin gel, high strength, compressive strength, rheological properties

## Abstract

Thermosetting resins have good temperature resistance and high strength and have been widely used as plugging agents in oil fields. However, the current resin materials have high costs, and unmodified thermosetting resins are brittle or have deteriorated properties such as flame retardancy after curing to form a crosslinked network structure. In this study, the resin was modified via physical blending. The curing strength and temperature resistance were used as the main indicators. The resin matrix, curing agent, rheology modifier, and filling materials were modified and formulated optimally to form a high-strength resin gel plugging system. The resin gel system exhibited good fluidity and pumpability. When the shear rate was 200 s^−1^ at 25 °C, the initial viscosity was 300–400 mPa·s. The viscosity gradually decreased with increasing shear rate, and the apparent viscosity had good long-term stability at room temperature. A contamination test of different types of drilling fluids on the resin gel system showed that this system had good anti-contamination capability and could maintain a high curing strength even after being contaminated. At the same time, the system exhibited good plugging capability. A wedge-shaped fracture with an inlet size of 7 mm and an outlet size of 5 mm was plugged at 12.84 MPa for 10 min without leakage. A sand-filling pipe (with a diameter of 3.8 cm and pipe length of 30 cm) connected to the pipeline with a 6 mm outlet was subjected to a constant pressure of 11.29 MPa and plugged for 8 min before breaking through. Therefore, it exhibited good capability for plugging fissures and cavities. The resin gel leakage-plugging system has significant potential to realize effective plugging of the deep large-fracture leakage layer.

## 1. Introduction

Lost circulation is a common problem in the drilling process of petroleum engineering. Improper selection of drilling location, unsuitability of drilling technology, and poor formation conditions are all causes of lost circulation [1,2]. For oil wells that still have economic value for exploitation, lost circulation can result in serious economic losses. In terms of construction progress and safety, lost circulation can prolong the operation cycle and lead to complex events such as kicks, blowouts, collapses, and drilling bit jamming [3,4].

Researchers have devoted tremendous efforts to solving lost circulation and have found that plugging materials are the key. Common plugging materials include cement slurries [5], high water-loss plugging materials, and polymer gels [6,7]. Cement slurries and high water-loss plugging materials are rigid plugging materials that rely on bridge plugs to realize the sealing of the leakage layer. Owing to their low price and good temperature resistance, rigid plugging materials have been widely used in the field [8]. However, there are often certain limitations in their field application. When encountering a large number of large cracks during plugging, it is not possible to form an effective plugging layer around the wellbore. Consequently, false plugging can easily occur. After resumption of drilling, the false plugging layer is prone to failure under the agitation of the drilling tool. Cement slurries are not prone to false plugging, but their resistance to contamination is poor. Moreover, they are prone to solidification failure under the dilution of formation fluids, resulting in plugging failure. Polymer gel is a new type of plugging material that has emerged in recent years, known as a flexible plugging material [9,10]. It mostly has a three-dimensional network produced by crosslinking polymers. Polymer gel has good water solubility and deformability owing to its many network structures and hydrophilic groups, such as carboxyl and hydroxyl groups. Thus, it can be applied to the deep bottom layer for plugging. Most gel plugging materials have a controllable curing time, which can result in accurate plugging of target layers. However, gel materials often have low strength and poor temperature resistance and cannot achieve high strength plugging of deep and large fracture formations [11,12].

Gel material is a kind of special semi-solid material formed by polymer material and liquid material under specific conditions [13]. Specifically, the gel material is composed of polymer solution or sol under certain conditions, through the polymer or colloid linear connection to form a spatial network structure. These structures give the gel material elastic and highly dispersed properties. Resin materials are polymer materials with three-dimensional crosslinked network structures. They have a higher strength and better temperature resistance than gel materials [14,15]. Resins are mainly classified into thermosetting and thermoplastic resins. Thermoplastic resins are materials that can be repeatedly heated and molded. When heated, they soften and become moldable; when cooled, they harden again but retain their previous shape. Thermosetting resin refers to a large class of synthetic resins that undergo chemical reactions under heating or pressure, or under the action of curing agents, etc., and are crosslinked to become insoluble and insoluble substances. This resin is generally a solid or viscous liquid with low molecular weight before curing. In the molding process, it can be softened or flow, with plasticity, and can be made into a certain shape, and at the same time, chemical reactions and crosslinking curing can occur. Once cured, it is impossible to soften or flow again under pressure heating. Its crosslinked curing reaction is irreversible, giving the material high strength, good heat resistance, corrosion resistance, aging resistance, good dimensional stability, and other excellent properties. Thermosetting resins are suitable for preparation into plugging agents. According to their main chemical compositions, commonly used thermosetting resins can be classified into epoxy [16], phenolic [17,18], unsaturated polyester, cyanate ester [19], polyimide [20], bismaleimide [21], and urea–formaldehyde resins [22]. Thermosetting resins are widely used for their good heat resistance, pressure resistance, and excellent mechanical properties due to their body structure.

However, most unmodified thermosetting resins are brittle or have deteriorated properties such as flame retardancy after curing to form a crosslinked network structure. Thermosetting resin modification is generally performed via physical blending or chemical crosslinking. At present, the modification of thermosetting resin mainly focuses on toughening, heat resistance, and flame retardancy modification. The molecular chains of thermosetting resin are crosslinked to form network structures by adding physical dispersed particles or chemical modifiers [5,23,24]. At high temperatures, a series of chemical reactions produces synergistic effects that significantly improve the toughness and heat resistance of materials. Modification of thermosetting resins by introducing flame retardant elements or functional groups through in situ polymerization or pre-polymerization improves their brittleness while increasing their thermal stability. Thermosetting resins with excellent thermal stability can be obtained by modifying them in different ways. Owing to the diversity of resin materials and their excellent bonding, heat resistance, pressure resistance, and mechanical properties after modification, they can adapt to different formation temperatures and pressures. The curing time and temperature can be controlled using additives such as curing agents, catalysts, and diluents. Resin gel is a modified resin that is usually obtained by mixing a synthetic resin (for example, epoxy resin) with a granular solid additive. It has high bonding strength and chemical resistance and is now widely used in concrete repair, ground hardening, and waterproof layer production [25]. Resin gel not only plugs fractures effectively, but also improves the overall durability and compressive strength of the structure. This excellent temperature resistance and strength makes it suitable for the preparation of plugging agents [26].

In this study, the resin was modified via physical blending. The curing strength and temperature resistance were used as the main indicators. The resin matrix, curing agent, rheology modifier, and filling materials were modified and formulated optimally to form a high-strength resin gel plugging system. Its curing mechanism was analyzed along with its rheology, contamination resistance, curing effect, thickening performance, and plugging performance. Combined with the application characteristics of high deformation ability before curing and high strength after curing, it can be predicted that the thermosetting resin has a broad application prospect in the field of plugging of fractured formation drilling fluids.

## 2. Results and Discussion

### 2.1. Research and Development of the Resin Gel Plugging System

#### 2.1.1. Resin Matrix Optimization

The resin matrix is the key factor in determining the curing strength, time, and temperature of the resin gel plugging system. Epoxy resin is a polymer prepolymer containing two or more epoxy groups; the main functional group is epoxy group (–O–CH_2_–CH–), these epoxy groups can react with other active functional groups (such as amines and phenols) to generate three-dimensional polymer network, thus giving it excellent physical and chemical properties. Urea–formaldehyde resin is a polymer compound formed by the condensation of urea and formaldehyde under the action of a catalyst. The main functional groups are derived from the reaction products of urea and formaldehyde, including hydroxymethyl (-CH_2_OH). The molecule contains a large number of hydroxymethyl and carbamate bonds, and these functional groups enable the resin to form a stable crosslinked structure during curing. Phenolic resin is a polymeric compound formed by the condensation of phenol and formaldehyde under the condition of a catalyst. The main functional groups are derived from the reaction products of phenol and formaldehyde, including hydroxymethyl and methylene. Its molecules contain a large number of hydroxymethyl and methylene functional groups; these functional groups make the resin have excellent high-temperature resistance. Based on the performance requirements of plugging materials for severe lost circulation plugging technology, we used three water-soluble resins, that is, epoxy, urea–formaldehyde, and phenolic resin, as the matrix, ammonium chloride as the curing agent, and deionized water as the solvent, stirring thoroughly to form resin gel. The mass fraction of epoxy resin, urea–formaldehyde resin and phenolic resin was 25%, and the mass fraction of the ammonium chloride curing agent was 5%. The resin quality to curing agent quality ratio was 5:1. The resin gel solution was cured at different temperatures, and its curing strength and curing time at different temperatures were evaluated, so as to select the best resin matrix. The experimental results are shown in Table 1.

According to the above experiments, the urea–formaldehyde resin cured well, whereas the phenolic and epoxy resins were liquid under the same conditions and did not undergo curing. Therefore, the curing effect of urea–formaldehyde resin at different temperatures was better than those of the other two resins; hence, urea–formaldehyde resin was chosen as the resin matrix in the preparation of the resin plugging agent.

The glass-transition temperatures of different resins were tested using differential scanning calorimetry (DSC), as shown in Table 2 [25], to further optimize the selection of different types of thermosetting resins. The experimental results showed that the urea–formaldehyde and epoxy resins were the best choices. The glass-transition temperature of the industrial urea–formaldehyde resin was found to be 163 °C using DSC. Considering that the formation temperature of the Tahe oil field is 120–150 °C, which is similar to the glass-transition temperature of urea–formaldehyde resin, the preferred resin matrix is urea–formaldehyde resin.

#### 2.1.2. Preparation of Water-Soluble Resin

**Figure 1:**
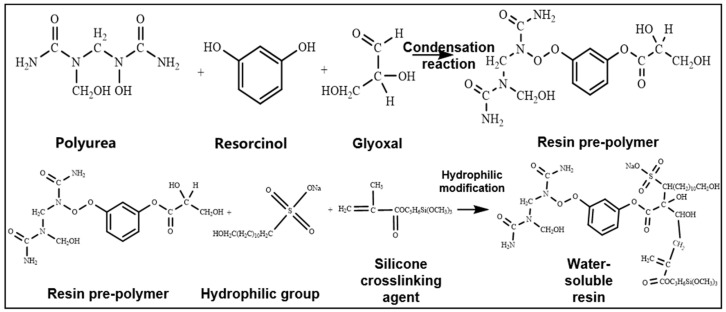
Reaction equation for the preparation of water-soluble resin.

**Figure 2:**
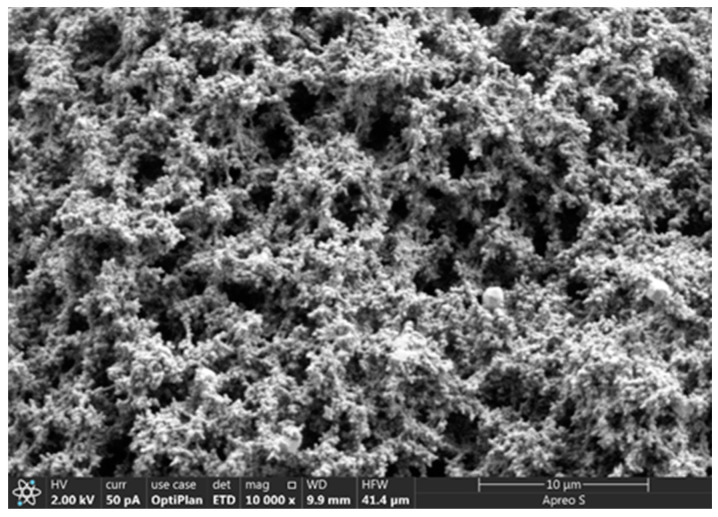
Microstructure of the water-soluble resin.

The resin system prepared with urea–formaldehyde resin as the matrix has good curing strength, proper glass transition temperature, and proper curing time, but its water solubility and dispersibility are poor, which greatly increases the difficulty of preparing resin mortar. The modification of resin matrix to prepare hydrophilic water-soluble resin can optimize its dispersibility and reduce the difficulty of preparation. The rheology and curing properties of water-soluble resin solutions can be easily adjusted using additives. Therefore, urea–formaldehyde resin was selected as the resin matrix in this study. The synthesis process was as follows: First, urea and formaldehyde were mixed evenly with resorcinol and furfural in a certain molar ratio (3:1). The resin prepolymer was then prepared via the polycondensation reaction after adjusting the pH value of the mixed solution to an acidic pH (pH = 4–6). Furthermore, the water-soluble modification of urea–formaldehyde resin is mainly through the internal emulsification method to directly introduce hydrophilic groups or chain extenders containing hydrophilic groups into the urea–formaldehyde resin prepolymer for chemical modification. After the prepared urea–formaldehyde resin is dissolved in water, the hydrophilic groups in the molecules are oriented towards the water phase, which can make it statically dispersed in water. A crosslinker was then added to the aqueous solution. When curing, the active groups contained in the resin, such as methylol, amide bond, and dimethyl ether bond, crosslinked with formaldehyde to form water-soluble resins with chain or network structures (Figure 1). Low-molecular-weight organosilicon compounds with special structures were selected as the resin crosslinker, which contained active functional groups such as epoxy, vinyl, acylamino, and alkoxy groups. One end of the crosslinker can react with silanol groups on the surface of inorganic materials such as glass fibers, silicates, and metal oxides to form covalent bonds. The other end can also form covalent bonds with the resin, thereby crosslinking two incompatible materials to form a network structure (Figure 2).

The optimal preparation conditions were optimized by studying the dosage of different chemical agents used in the preparation of water-soluble resins through orthogonal experiments. The crosslinking temperature was 40 °C, the reaction time was 4 h, and the total content of urea–formaldehyde resin and other additives was 100%. The orthogonal optimization test scheme is shown in Table 3. The results of the study showed that Formulation 7 had the longest sedimentation time (168 h). Thus, the optimal preparation conditions for the water-soluble resin were as follows: 0.6% resorcinol, 1.5% furfural, 0.9% sodium dodecyl sulfate, 0.5% organosilicon crosslinker, and the remainder was the urea–formaldehyde resin.

#### 2.1.3. Curing Agent Optimization

A latent curing agent is a special type of curing agent that triggers a chemical reaction to cure a resin only under specific conditions. This curing agent is usually inactive under normal storage conditions and will only begin the curing process when specific conditions are met, providing better long-term stability and controlled curing time. Traditional curing agents have a short curing time. To extend the curing time of the resin gel system, a latent curing agent was used to prepare and optimize the resin aqueous solution. The curing effect was also tested (at a curing temperature of 120 °C and curing time of 130 min).

**Figure 3:**
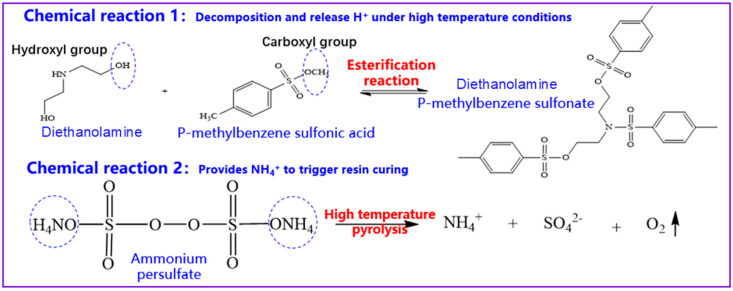
Reaction equation of the latent curing agent control mechanism.

Figure 3 shows the curing mechanism of the curing agent. The latent curing agent used in this study consisted of p-toluene sulfonic acid, hexamethylenetetramine, diethanolamine, and ammonium persulfate. The latent curing agent is a substance that does not exhibit acidity at low temperatures but exhibits acidity at high temperatures. It can have an inhibitory effect on the precure behavior and control the curing performance of urea–formaldehyde resin. P-toluene sulfonic acid and diethanolamine in the latent curing agent produced an esterification reaction to generate a strong-acid weak-base salt. High-temperature decomposition released H^+^. Thus, the solution was acidic, and curing was triggered. The high-temperature pyrolysis of ammonium persulfate generated NH_4_^+^, which triggered the curing of water-soluble resins, leading to crosslinking curing of carbonyl ŀ“groups in free formaldehyde in the resin to form a three-dimensional network structure.

**Figure 4:**
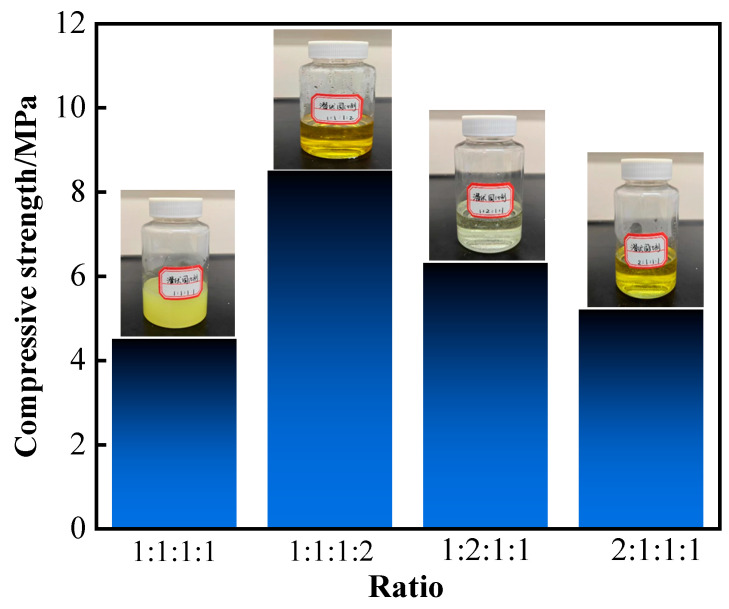
Latent curing agents with different proportioning ratios.

In this study, we optimized the proportioning of latent curing agents and studied the effects of different proportions on the curing effect of the resin gel system after hot rolling at 120 °C for 130 min. The proportioning of p-toluene sulfonic acid: hexamethylenetetramine: diethanolamine: ammonium persulfate in the latent curing agent was 1:1:1:1, 1:1:1:2, 1:2:1:1, and 2:1:1:1. The results showed that the highest strength of the cement formed by the curing of the resin gel system was achieved when the proportioning of the latent curing agent (p-toluene sulfonic acid: hexamethylenetetramine: diethanolamine: ammonium persulfate) was 1:1:1:2, with a compressive strength of 8.5 MPa (Figure 4). When the proportioning of the latent curing agent (p-toluene sulfonic acid: hexamethylenetetramine: diethanolamine: ammonium persulfate) was 1:1:1:1, 1:2:1:1, and 2:1:1:1, the resin gel system could also be cured to form a solidified body; however, the strength was not high. The reason was that when the proportioning of the latent curing agent (p-toluene sulfonic acid: hexamethylenetetramine: diethanolamine: ammonium persulfate) was 1:1:1:2, the content of p-toluene sulfonic acid was low, whereas the content of ammonium persulfate was relatively high. When the latent curing agent was mixed with urea–formaldehyde resin, it maintained a certain stability at room temperature and did not immediately react with the resin. However, when subjected to external stimuli, such as heat and light, the reactivity of the latent curing agent was released. During the curing process, the active groups in the latent curing agent react with the methylol or methylene in the urea–formaldehyde resin to form a crosslinked structure. This crosslinking reaction causes the urea–formaldehyde resin to transform from a linear to a bulk structure, which leads to curing. The cured urea–formaldehyde resin has high strength and hardness to meet specific usage requirements.

#### 2.1.4. Research and Development of Resin Plugging Materials

The synthesis process of urea–formaldehyde resins mainly consists of two stages: addition and polycondensation reactions. The first stage is the addition reaction. In a neutral or weakly alkaline environment (pH = 7–8), formaldehyde and urea undergoes a hydroxymethylation reaction dominated by polycondensation intermediates such as monohydroxymethylurea and dihydroxymethylurea. The type and amount of polycondensation intermediates can vary depending on the molar ratio of urea to formaldehyde. According to the principle of the steric hindrance of chemical functional groups, the greater the number of hydroxymethyl groups, the lower the capability of the remaining hydrogen atoms on the amino group of urea to undergo addition and polycondensation. The ratio of the rate of formation of monohydroxymethylurea and dihydroxymethylurea from the reaction between formaldehyde and urea is 9:3. The second stage is the polycondensation reaction under acidic conditions with a pH value of 4–6. The methylol groups on the hydroxymethylurea molecule crosslink to form a polymer with a linear or network structure. These linear and branched polymers can generate three-dimensional network products during resin curing. Finally, polymethylene urea that is insoluble in water and organic matter can be obtained, and a further reaction can form a network structure of urea–formaldehyde resin. Based on the principles of physical blending and chemical modification reaction, high-temperature controllable curing resin materials were prepared using the solution stepwise ring-opening polymerization method with water-soluble urea–formaldehyde resin as the matrix and the latent curing agent mixed together.

**Figure 5:**
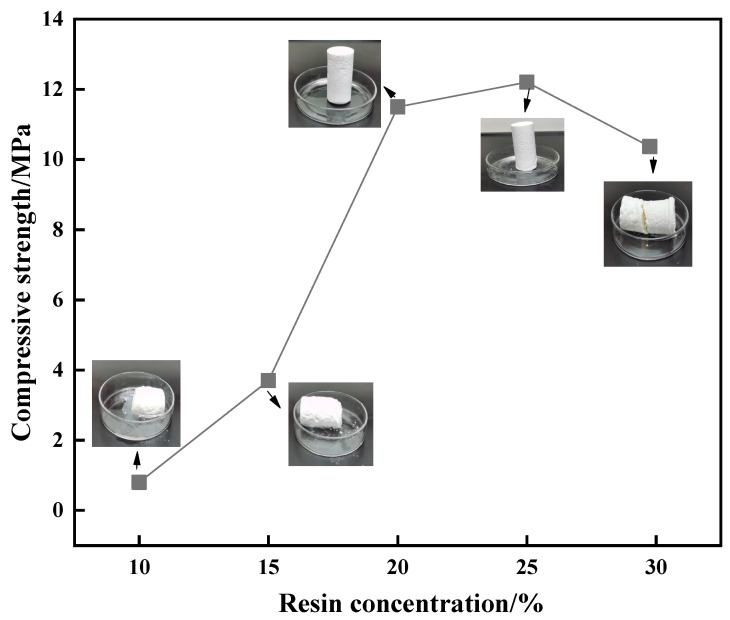
Effect of resin concentration on curing strength.

To optimize the resin curing effect and reduce costs, a single factor analysis experiment was conducted, as shown in Figure 5. The formula of the resin plugging system was optimized by changing the dosage of the urea–formaldehyde resin matrix (resin dosages of 10%, 15%, 20%, 25%, and 30%, and curing agent content fixed at 1%). The curing temperature and time were set to 130 °C and 180 min, respectively, to observe the curing effect. The experimental results showed that when the urea–formaldehyde resin was added at 10% and 15%, its curing effect was poor. Initial curing occurred, but the curing strength was small. When the urea–formaldehyde resin was added at 20% and 25%, its curing effect was the best. The curing strength was higher when it was cured into a cylindrical solid body. When the urea–formaldehyde resin was added at 30%, its curing effect was weakened again, and the sample was fractured after curing. The curing strength decreased. Therefore, the best curing effect was achieved when the urea–formaldehyde resin dosage was 20%.

**Figure 6:**
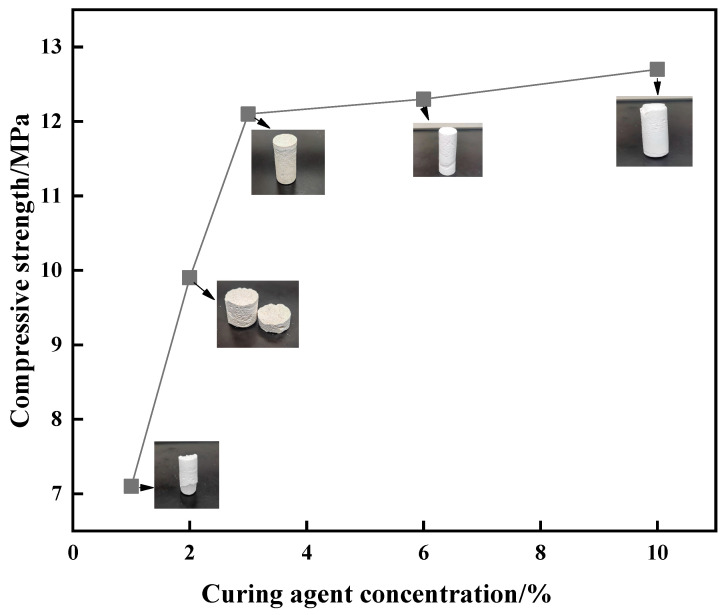
Effect of curing agent concentration on curing strength.

To optimize the dosage of the curing agent, single factor analysis experiments were conducted (curing agent dosages of 1%, 2%, 3%, 6%, and 10%, and fixed resin dosage of 20%). As shown in Figure 6, the curing strength increased and then decreased with increasing resin concentration. When the concentration of the urea–formaldehyde resin was 20%, the compressive strength was 11 MPa. When the concentration was 25%, the compressive strength reached 12 MPa. When the concentration was further increased to 30%, the compressive strength was reduced to 10.1 MPa. To further study the factors affecting the curing strength of the resin, compressive strength tests were performed on resin samples formed with different concentrations of the curing agent. The curing strength of the resin first increased and then stabilized with increasing curing agent concentration. When the curing agent concentration was 3%, the curing strength was at the turning point. An increase in curing strength was not obvious with further increase in curing agent concentration.

Single factor analysis was performed through orthogonal experiments. The dosage of resin, curing agent, etc., was varied, and considering the economic cost, the formula and proportion of the resin leakage-plugging material were determined as 20% water-soluble resin + 3% latent curing agent. The curing temperature (100–140 °C), time (120–300 min), and strength (10–18 MPa) could be controlled by adjusting the concentrations of the resin and curing agent.

#### 2.1.5. Preparation of the Rheology Modifier

Resin gel requires the addition of filling materials. However, simply adding filling materials will cause rapid sedimentation, resulting in a poor curing effect or even curing failure. Bentonite has good suspension and rheological modification capabilities. The suspension capacity of bentonite is mainly reflected in two parameters: colloid value and swelling volume. For bentonite of the same type, the more montmorillonite it contains, the higher its swelling volume and colloid value. The colloid value is the volume of the gel formed by mixing bentonite with water in proportion and adding an appropriate amount of magnesium oxide. It is generally expressed as the number of milliliters of the gel volume formed by 15 g of sample. This value reflects the degree of dispersion and hydration of the sample particles, and is a comprehensive expression of the dispersion, hydrophilicity, and expansibility of bentonite. The size of gum price is closely related to the type of bentonite ore and the content of montmorillonite. In general, the colloid value of sodium bentonite is higher than that of calcium bentonite and acid bentonite. For bentonite of the same genus type, the more montmorillonite contained, the higher the colloid value. Traditional bentonite is mostly calcium based, and its performance is not sufficiently stable. Lithium-based bentonite has good suspension properties, high swelling volume, and good adsorption properties [27]. Therefore, this study conducted lithiation modification on natural calcium-based bentonite.

In this experiment, the effect of the mass ratio of Li_2_CO_3_ to H_2_C_2_O_4_ on the modification was studied using purified bentonite as the raw material and the single factor analysis method. The swelling volume and colloid value were used as the basis for evaluating the effect and to determine the optimal lithiation conditions. The cations in montmorillonite have an exchange capacity. In this study, Li_2_CO_3_ was selected as a modifier, and Li^+^ was used to replace the exchangeable cations (Ca^2+^, Mg^2+^, etc.) in the bentonite to prepare lithium-based bentonite.

**Figure 7:**
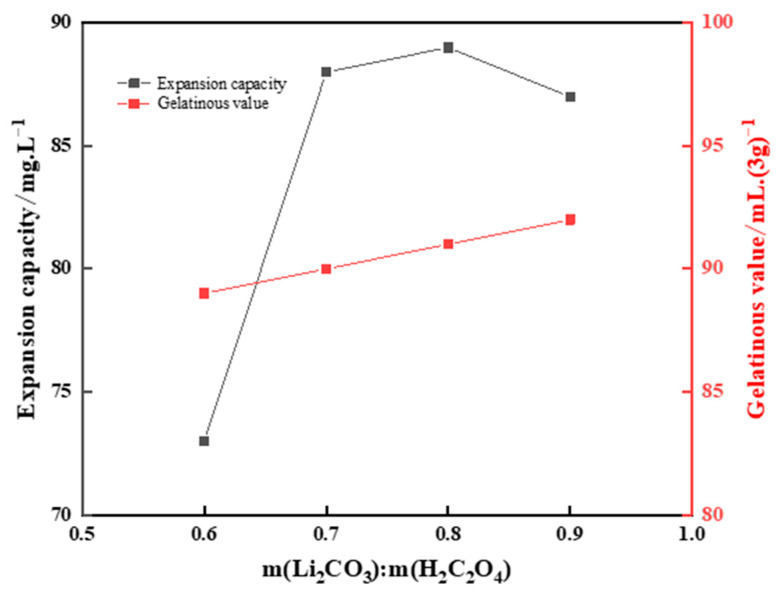
Effect of the Li_2_CO_3_:H_2_C_2_O_4_ ratio on the effect of lithiation modification.

Five grams of purified bentonite was weighed and placed in a beaker, and 100 mL of distilled water was added to prepare a bentonite dispersion with 5% concentration. Mixed solutions of (m(Li_2_CO_3_):m(H_2_C_2_O_4_)) were poured in different proportions into a beaker and stirred for 1.5 h in a constant-temperature water bath (T = 80 °C). The pH value of the solution system was controlled between 7 and 8. The effect of the Li_2_CO_3_/H_2_C_2_O_4_ ratio on the effect of lithiation modification is shown in Figure 7.

Figure 7 shows that with an increase in the ratio of Li_2_CO_3_ to H_2_C_2_O_4_, the swelling volume of bentonite also gradually increased. When the ratio reached 0.8, the swelling volume of bentonite reached its maximum value. When the ratio was more than 0.8, the swelling volume decreased. The reason for this may be that Li^+^ not only exchanged ions with interlayer adsorbed cations, but also entered the lattice to replace cations in the octahedron, causing lattice distortion and further reducing the swelling volume of modified bentonite. The colloid value of bentonite increased slightly with an increase in the ratio, probably because excessive Li^+^ hindered the exchange of ions to such an extent that the colloid value did not change significantly. Therefore, m(Li_2_CO_3_):m(H_2_C_2_O_4_) = 0.8 was chosen for this experiment.

The finalized bentonite modification conditions were as follows: A mass ratio of Li_2_CO_3_ to H_2_C_2_O_4_ of 0.8, bentonite dispersion concentration of 5%, constant-temperature reaction at 80 °C for 1.5 h, and a pH value of 7–8 for the solution system.

#### 2.1.6. Filling Material Optimization

The filling material is a solid material used to change the properties of the object or reduce the cost. To reduce the cost of the controllable curing resin and increase its curing strength, the preferred filling materials are quartz sand, barite, walnut shells, nano-silica, and fibers, which can be stabilized and dispersed in the resin solution with the help of a rheology modifier. The curing strength of the resin can be increased.

**Figure 8:**
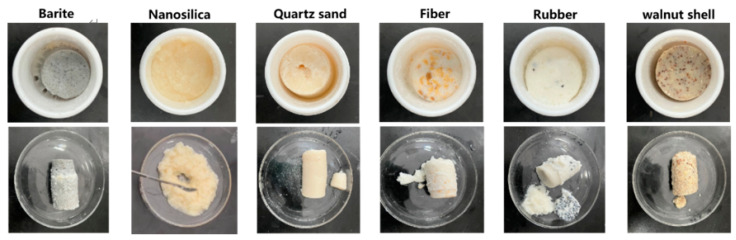
Curing effect of different filling materials added to the resin.

Walnut shells, nano-silica, quartz sand, and other filling materials were added to the resin solution, and the curing effect was observed. As shown in Figure 8, the addition of nano-silica, walnut shells, and quartz sand helped the resin plugging material to solidify and form a solid body of higher strength. Considering the impact of density on wellbore safety and stability, a combination of nano-silica, walnut shell, and quartz sand was initially selected as the optimal filling material. The mass ratio of nano-silica, walnut shell, and quartz sand is 1:1:1.

**Figure 9:**
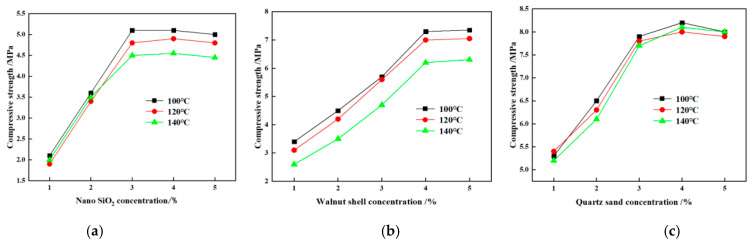
Effect of filling material dosage on curing strength: (**a**) nano-silica, (**b**) walnut shell, and (**c**) quartz sand.

Walnut shell is a natural filter material, its surface has many micropores, and the adsorption effect is good, but the specific surface area data vary according to the processing method and particle size. The particle size of walnut shell used in this study is 40 mesh. The particle size of nano-silica is very small, usually between 10–20 nm, or even smaller, and the particle size of nano-silica used in this study is 20 nm. Due to the extremely small particle size, the specific surface area of nano-silica is extremely large, usually up to hundreds of square meters per gram. Quartz sand has a wide range of particle sizes, ranging from 0.020 mm to 3.350 mm according to different mining and processing methods. Common specifications include 2–4 mesh, 4–6 mesh, and 6–10 mesh. The particle size of quartz sand used in this study is 160 mesh. The above combination was selected as the filling material for the experiment, and a hot-roll-curing experiment was conducted at different temperatures (100 °C, 120 °C, and 140 °C). As shown in Figure 9, the proportioning of the filling material was chosen according to its curing strength. As preferred, nano-silica was used in a proportion of 3%, walnut shells were used in a proportion of 4%, and quartz sand was used in a proportion of 3%.

**Figure 10:**
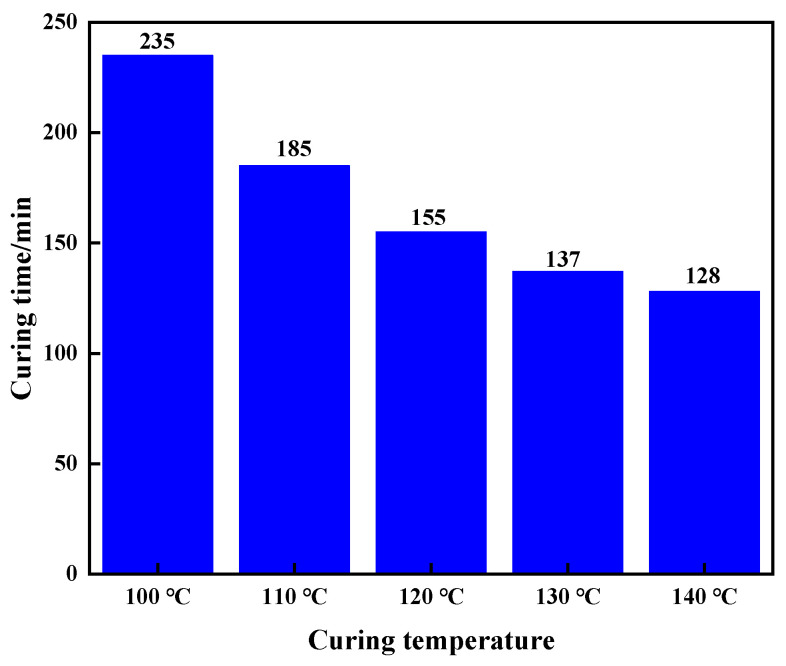
Effect of temperature on curing time.

The filling material (3% nano-silica + 4% walnut shell + 3% quartz sand with a density of 1.11 g/cm^3^) was selected for hot-rolling experiments at 100 °C, 120 °C, and 140 °C to observe the curing time and strength. As shown in Figure 10, the curing time was 235 min at 100 °C and 128 min at 140 °C. The system formulation was determined as 20% urea–formaldehyde resin + 1% rheology modifier + 3% curing agent + 10% filling material.

### 2.2. Performance of the Resin Gel Plugging System

#### 2.2.1. Rheological Properties of the Resin Gel Plugging System

**Figure 11:**
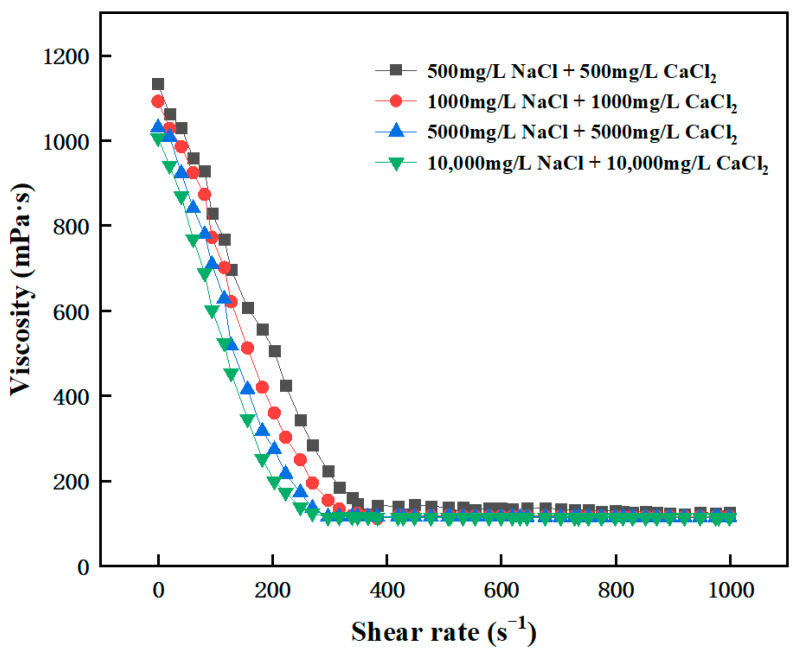
Variation in the viscosity with shear rate for the resin gel system.

Viscosity is an important parameter for characterizing the rheological properties. The rheological properties of high-temperature-resistant and high-strength curable resins prior to curing were tested using a HAAKE MARS Model 60 high-temperature, high-pressure rotational rheometer. The variation in viscosity with shear rate of this resin solution before curing was measured using the HAAKE rheometer. The initial viscosity of the resin gel system was between 300 and 400 mPa·s. The viscosity gradually decreased with increasing shear rate and eventually stabilized (Figure 11). The resin gel system had the characteristic of “shear thinning”, which was conducive to on-site pumping and did not require the use of high-pressure pump trucks for pumping.

**Figure 12:**
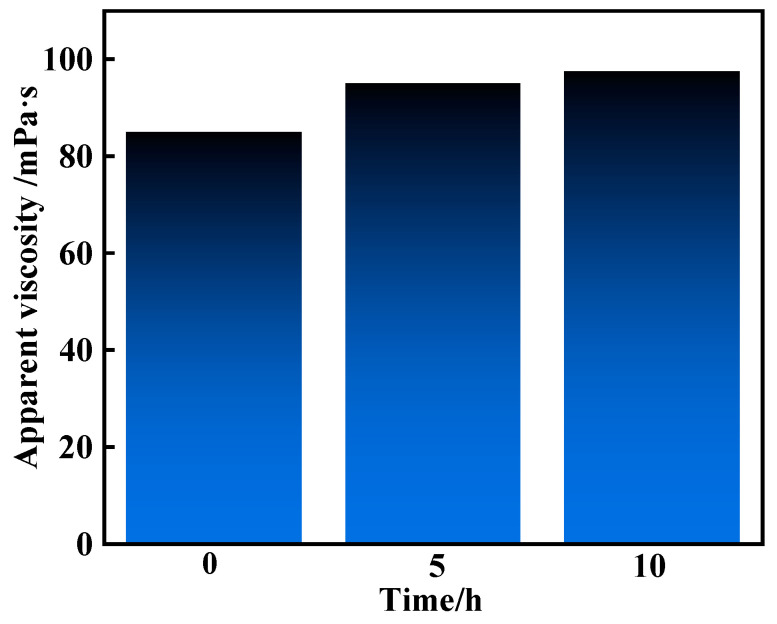
Rheology of the resin gel system after mixing for different times at room temperature.

To study the rheology of the resin gel system with different mixing times at room temperature, the solution state of the system in the process of floor mixing was simulated. The shear rate was 300 s^−1^ and the experimental temperature was 25 °C. The results (Figure 12) showed that the initial apparent viscosity of the resin gel system was 85 mPa·s, which became 95 mPa·s after 5 h of mixing, and 97.5 mPa·s after 10 h of mixing. The apparent viscosity of the resin gel system was relatively stable at room temperature, without thickening. The resin gel system did not solidify in the slurry tank.

**Figure 13:**
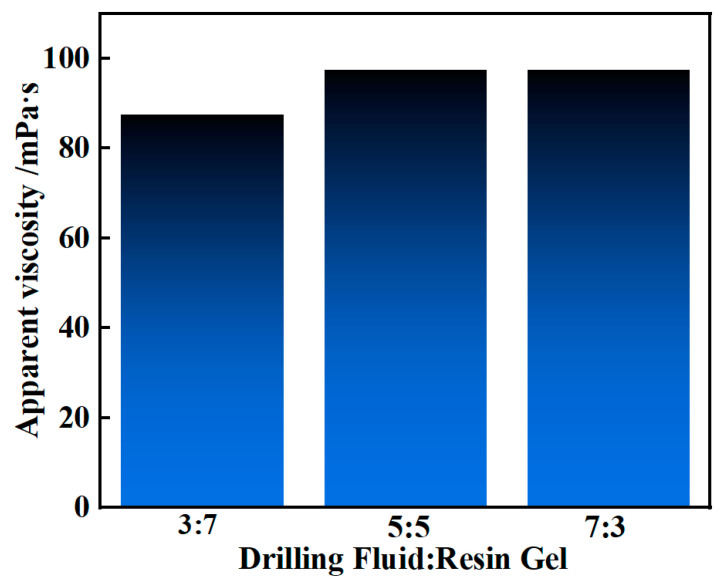
Effect of different drilling fluid dosages on the rheology of the resin slurry system.

Drilling fluid and resin gel were mixed in proportions of 3:7, 5:5, and 7:3 to study the effect of drilling fluid addition on the rheology of the resin gel system. As shown in Figure 13, the addition of drilling fluid influenced the rheology of the system. The apparent viscosity of the resin gel system itself was 85 mPa·s. It slightly increased to 87.5 mPa·s, 97.5 mPa·s, and 97.5 mPa·s owing to hydration after the addition of drilling fluids in the proportions of 3:7, 5:5, and 7:3, respectively.

#### 2.2.2. Influence of Drilling Fluid Contamination on the Curing Effect of the Resin Gel System

**Figure 14:**
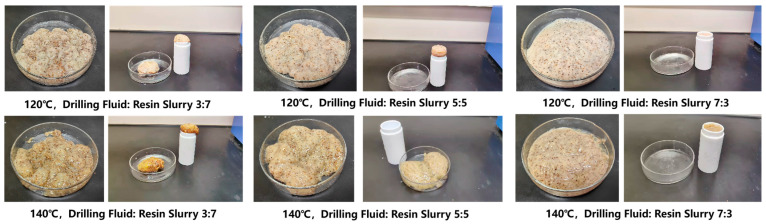
Influence of drilling fluid addition on the curing effect of the resin gel system.

As shown in Figure 14, the drilling fluid was mixed with resin gel in proportions of 3:7, 5:5, and 7:3. After stirring evenly, the curing effect was observed by hot rolling at 120 °C and 140 °C for 2.5 h. Mixing the drilling fluid with resin gel in the proportions of 3:7, 5:5, and 7:3 did not cause flash solidification but formed only a viscous colloid. It was unable to form a solidified body with a certain strength. Therefore, it is necessary to avoid mixing drilling fluid and resin gel during on-site construction and to consider injecting isolation fluid in advance.

**Figure 15:**
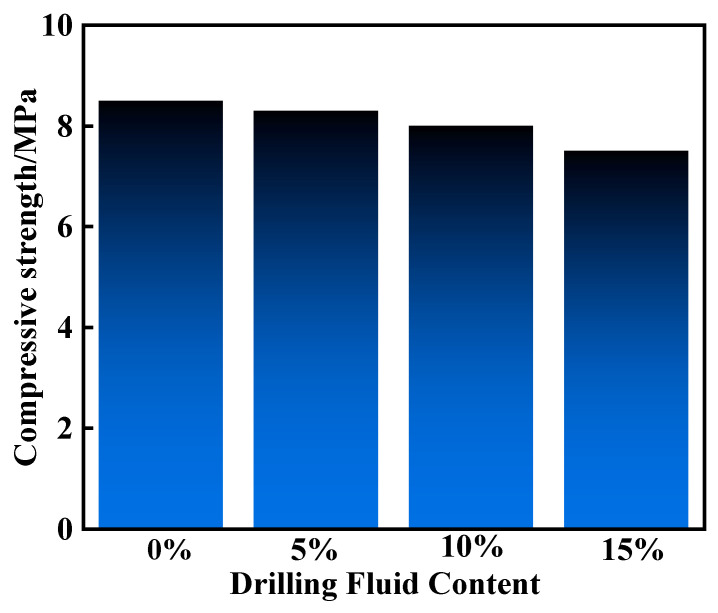
Compressive strength of the resin slurry system with added drilling fluid.

To further study the effect of drilling fluid addition on the curing effect of the resin gel system, 5%, 10%, and 15% drilling fluid were added sequentially to the resin gel system under hot rolling at 130 °C for 120 min. Figure 15 shows the curing compressive strength of the resin gel system with different amounts of drilling fluids added. As shown, the addition of a small amount of drilling fluid to the system had a small effect on the curing strength. As a result, some of the drilling fluid left in the slurry tank during on-site slurry preparation may affect its curing effect. Therefore, the slurry tank should be cleaned before slurry preparation or a freshwater tank should be used.

**Figure 16:**
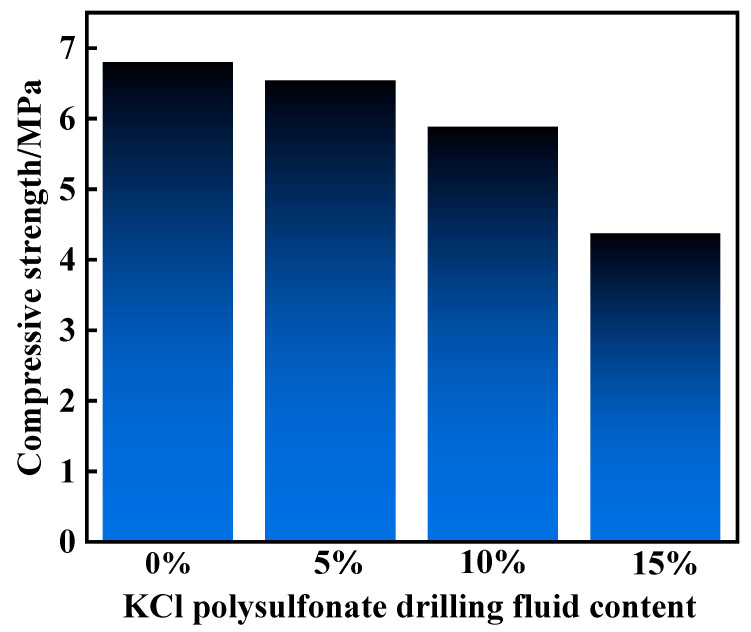
Curing effect of KCl polysulfonate drilling fluid on the resin gel system.

The curing effect of the resin gel plugging system with 10%, 15%, and 20% KCl polysulfonate drilling fluid added sequentially (Figure 16) was also investigated after hot rolling at 130 °C for 120 min. In the KCL polysulfonate drilling fluid system, the mass fraction of KCL is 5% and the mass fraction of polysulfonate is 12.5%. The results showed that the resin gel systems with 10% and 15% KCl polysulfonate drilling fluid were still able to cure into high-strength cement. The curing strength was slightly reduced with 20% KCl polysulfonate drilling fluid. The compressive strength of the cured sample was measured at 6.54 MPa, 5.88 MPa, and 4.37 MPa with the addition of 10%, 15%, and 20% KCl polysulfonate drilling fluid, respectively. Although the strength was slightly decreased, the compressive strength was still higher than 4 MPa with the addition of 20% KCl polysulfonate drilling fluid. This indicated that the resin gel system had a good resistance to KCL polysulfonate drilling fluid contamination.

#### 2.2.3. Evaluation of the Thickening Performance of the Resin Gel Plugging System

**Figure 17:**
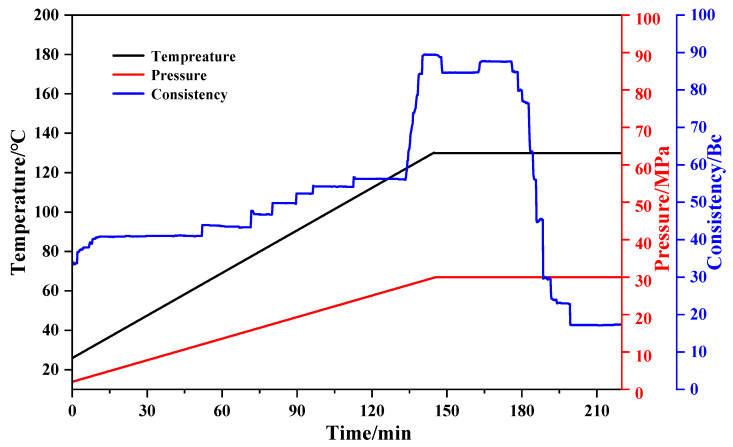
Thickening curve of the resin gel plugging system.

To clarify the adaptability of the resin gel for field application, the thickening performance of the resin gel plugging system was evaluated by setting the experimental temperature as 130 °C, the warming and thickening time as 140 min, and the pressure as 30 MPa. “Bc” stands for consistency, also commonly referred to as viscosity or viscosity, and is a physical quantity that describes the resistance of a fluid (liquid or gas) to fluidity. Specifically, it reflects the amount of internal friction in a fluid when subjected to shear forces. In other words, a fluid with a higher consistency requires more force to overcome the frictional resistance within it as it flows. Consistency is measured in centipoise (cP). As shown in Figure 17, with the increase in the test temperature and pressure, the consistency curve exhibited a stepwise increase. When the resin gel leakage-plugging system temperature increased to 130 °C and the pressure reached 30 MPa, the thickening time used was 140 min, and the consistency reached 89.1 Bc. With the increase in time, the consistency slightly decreased. At 180 min, the consistency instantly decreased to 18 Bc. At this time, the resin gel plugging system had been completely cured. Its state is shown in Figure 17. The figure shows that the initial curing of the resin gel plugging system occurred in 134 min, and it was fully cured at 180 min with a certain strength. However, the thickening mixing rod did not stick to the resin gel system. It also indicated that the resin gel plugging system had less adhesion to the drilling rod and did not cause difficulties in drilling.

#### 2.2.4. Evaluation of the Pressure-Bearing Plugging Performance of the Resin Gel Plugging System

**Figure 18:**
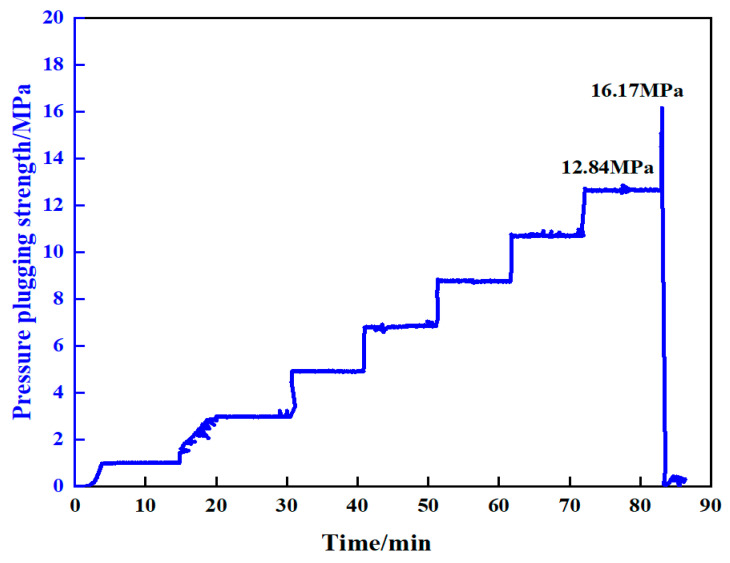
Pressure-bearing plugging capacity curve for wedge-shaped fractures (inlet 7 mm, outlet 5 mm).

The pressure-bearing plugging performance of resin gel system at 100 °C was evaluated using a high-temperature and high-pressure plugging simulation device. Curing was conducted at 100 °C for 220 min, with a pumping rate of 10 mL/min and plugging at a constant pressure for 10 min. The experimental results are shown in Figure 18. The resin gel plugging system showed good fracture plugging capability. A wedge-shaped fracture with an inlet size of 7 mm and an outlet size of 5 mm was plugged at 12.84 MPa for 10 min without leakage. The breakthrough occurred when the pressure was increased to 16.17 MPa.

**Figure 19:**
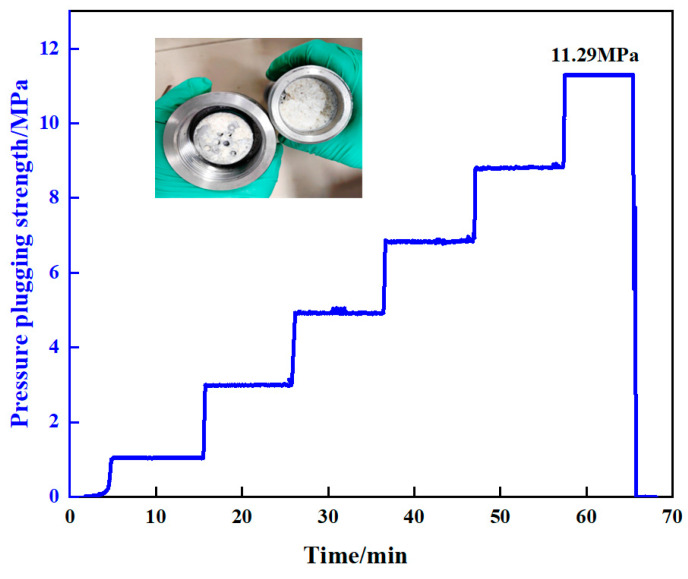
Pressurized plugging capacity curve of sand-filling pipe (with a diameter of 3.8 cm, pipe length of 30 cm).

A high-temperature and high-pressure plugging simulator was utilized to evaluate the pressure-bearing plugging performance of the resin gel system in sand-filling pipes at 100 °C. The experimental conditions were as follows: 100 °C curing for 220 min, pumping rate of 10 mL/min, and plugging for 10 min at a constant pressure at an interval of 2 MPa. The experimental results are shown in Figure 19. The sand-filling pipe (with a diameter of 3.8 cm and pipe length of 30 cm) connected to the pipeline with a 6 mm outlet was subjected to a constant pressure of 11.29 MPa and plugged for 8 min before breaking through. Therefore, the resin gel system had good capability for plugging fissures and cavities.

## 3. Conclusions

(1) In this paper, a resin plugging system based on urea–formaldehyde resin was prepared for plugging formation cracks. Using curing strength and curing time as the main indexes, the additive amount and types of various materials in the resin plugging system were optimized.
(2) The optimal ratio of latent curing agent combination is p-methylbenzenesulfonic acid: hexamethylenetetramine: diethanolamine: ammonium persulfate ratio = 1:1:1:2. The flow pattern regulator is a combination of Li_2_CO_3_ and H_2_C_2_O_4_ (m(Li_2_CO_3_):m(H_2_C_2_O_4_) = 0.8). The filling material is a combination of 3% nano-silica, 4% walnut shell, and 3% quartz sand, and the final formula of the system is: 20% urea formaldehyde resin + 1% rheological regulator + 3% curing agent + 10% filling material. The density is 1.11 g/cm^3^.
(3) The resin gel plugging system has good fluidity and pumpability, the initial viscosity is between 300–400 mPa·s, the viscosity gradually decreases with the increase in shear rate, and the apparent viscosity has good long-term stability at room temperature, and has the potential for field application.
(4) The resin gel plugging system has good anti-pollution and anti-dilution ability, and the curing strength can still be greater than 4 MPa at high temperature after the pollution of KCL polysulfonate drilling fluid.
(5) The resin gel leakage plugging system showed good plugging ability. The wedge crack with the inlet size of 7 mm and outlet size of 5 mm was plugged at a constant pressure of 12.84 MPa for 10 min without leakage; the sand filling pipe (diameter of 3.8 cm and pipe length of 30 cm) connected to the outlet 6 mm pipeline was plugged at a constant pressure of 10.73 MPa for 8 min and broke through. It indicates that it has good sealing ability.

## 4. Materials and Methods

### 4.1. Experimental Materials

Polymethylene urea is a linear oligomer obtained via the polycondensation of urea and formaldehyde solution under the catalytic effect. In this study, the resorcinol, furfural, sodium dodecyl sulfate, 3-(methacryloyloxy) propyl trimethoxysilane, p-toluene sulfonic acid, diethanolamine, and nano-silica were all analytical grade and purchased from Aladdin Chemical Reagent Co., Ltd. (Riverside, CA, USA).The ammonium chloride, sodium chloride, calcium chloride, ammonium persulfate, and diammonium hydrogen phosphate were all analytical grade and purchased from Sinopharm Group Chemical Reagent Co., Ltd. (Shanghai, China). The barite, quartz sand, and walnut shells were all industrial products purchased from Shandong Xiya Chemical Co., Ltd. (Binzhou, China). The rheology modifier was purchased from Shandong Shida Oilfield Technology Service Co., Ltd. (Dongying, China).

### 4.2. Experimental Methods

#### 4.2.1. Rheology

The rheological properties of the sample solutions before curing using the resin gel plugging system were tested using a HAAKE MARS 60 rotational rheometer (Thermo Fisher Scientific, Waltham, MA, USA). The rotor model used for the experiment was CC41/Ti (with a rotor diameter of 41 mm); the rheometer measures the cylinder diameter of 48 mm [28]. The test sample temperature was equilibrated for at least 30 min, and the temperature error was controlled to ±0.1 °C. The viscosity of the sample solutions at different shear rates before curing using the resin gel plugging system were measured [29,30].

The colloid value refers to the volume of gel formed by mixing bentonite and water in proportion and adding an appropriate amount of magnesium oxide to induce agglomeration. The colloid value and swelling volume of modified bentonite were used as the basis for evaluating the modification effect. The colloid value represents the dispersion and hydration degree of the sample particles and is a comprehensive manifestation of dispersibility, hydrophilicity, and expansibility. Its magnitude is closely related to the type of bentonite ore and the content of montmorillonite. Sodium-based bentonite has a higher colloid value than calcium-based and acidic bentonite. For bentonite of the same type, the more montmorillonite it contains, the higher its colloid value. Therefore, the colloid value is one of the technical indicators for identifying the type of bentonite ore and estimating the quality of bentonite. The swelling volume refers to the volume of bentonite in diluted hydrochloric acid solution after expansion. It is also a technical indicator for identifying the type of bentonite ore and estimating the quality of bentonite, expressed in ml/g. Similarly, sodium-based bentonite has a higher swelling volume than calcium-based and acidic bentonite. For bentonite of the same type, the more montmorillonite it contains, the higher its swelling volume.

#### 4.2.2. Microstructural Characterization

A Hitachi S-4700 field-emission scanning electron microscope (SEM) (Hitachi of Japan, Tokyo, Japan) was used to characterize the microstructure of the cured samples. The treated samples were carefully cut off to obtain a new cross-section, which was then mounted on an aluminum root and sprayed with a thin layer of gold for scanning. Scanning electron microscopy imaging was then performed at 10 kV.

#### 4.2.3. Sedimentation Test

The water-soluble sample was left to stand at room temperature and observed for sedimentation or stratification every 12 h. When sedimentation or stratification occurred, the water-soluble sample was considered to have failed, and the time at this point was the failure time.

#### 4.2.4. Compressive Strength

The sample cured by the resin gel plugging system was formed into a cylindrical shape with a diameter of 10 mm and a height of 10 mm. The compressive mechanical properties were tested at room temperature using an electronic universal testing machine (CMT4000 electronic universal testing machine, Shenzhen Newsansi Material Testing Company, Shenzhen, China). The compression speed was set to 20 mm/min, and the stress–strain curve of the sample was recorded under compression.

#### 4.2.5. Contamination Resistance

Polymer drilling fluid and polysulfonate drilling fluid with volumes of 5%, 10%, and 15% were added to the resin gel plugging system, and the curing effect of the system was observed after hot rolling at 140 °C for 150 min. The curing strength was tested after adding the polymer and polysulfonate drilling fluids separately, and their contamination resistance performances were clarified.

#### 4.2.6. Plugging Performance

A physical simulation device for high-temperature and high-pressure fracture plugging was used to study the plugging performance of the resin gel sealing system on fractures. Additionally, a physical simulation device for high-temperature and high-pressure sand filling tubes was used to study the plugging performance of the system on fissures and cavities. The resin gel plugging system was injected into the fractured core and steel sand-filling pipe and cured at 140 °C for 200 min. The simulated formation water was injected into the fractured core and sand-filling pipe at an injection rate of 10.0 mL/min. Plugging was performed at a constant pressure for 10 min at intervals of 2 MPa. The change in the injected pressure was recorded in real time using the data software, and the highest recorded pressure was considered the pressure-bearing plugging capacity of the resin gel system.

## Figures and Tables

**Table 1:** Curing of different resin matrices.

Curing Time and Strength	45 °C	60 °C	80 °C	100 °C
Urea–formaldehyde resin	Uncured	1 h, moderate strength	2 h, high strength	3.46 h, high strength
Phenolic resin	Uncured	Yellow emulsion	Light yellow liquid	Yellow liquid, aqueous
Epoxy resin	Uncured	Clear liquid with precipitation	Aqueous transparent liquid	Aqueous transparent liquid

**Table 2:** Glass-transition temperatures of different resin.

Resin Type	Glass-Transition Temperature (Tg)/°C
Phenolic resin (PF)	85–100 °C
Epoxy resin (EP)	>105 °C
Bismaleimide resin (BMI)	177–232 °C
Unsaturated polyester resin (UPR)	50–60 °C
Cyanate resin (CE)	240–260 °C
Polyimide resin (PR)	>300 °C
Urea–formaldehyde resin (UF)	>160 °C

**Table 3:** Orthogonal optimization test for water-soluble resin preparation.

	Urea–Formaldehyde Resin/%	Resorcinol %	Furfural %	Sodium Dodecyl Sulfate %	Organosilicon Crosslinker	Sedimentation Time/h
1	Remainder is urea–formaldehyde resin	0.3	0.5	0.3	0.1	36
2		0.3	1.0	0.6	0.3	72
3		0.3	1.5	0.9	0.5	72
4		0.3	2.0	1.2	0.7	120
5		0.6	0.5	0.3	0.1	132
6		0.6	1.0	0.6	0.3	144
7		0.6	1.5	0.9	0.5	168
8		0.6	2.0	1.2	0.7	84
9		0.9	0.5	0.3	0.1	48
10		0.9	1.0	0.6	0.3	72
11		0.9	1.5	0.9	0.5	84
12		0.9	2.0	1.2	0.7	60
13		1.2	0.5	0.3	0.1	36
12		1.2	1.0	0.6	0.3	144
15		1.2	1.5	0.9	0.5	84
16		1.2	2.0	1.2	0.7	134

## Data Availability

The original contributions presented in the study are included in the article; further inquiries can be directed to the corresponding author.
